# Mistrust among Minorities and the Trustworthiness of Medicine

**DOI:** 10.1371/journal.pmed.0030244

**Published:** 2006-05-30

**Authors:** Matthew K Wynia, Vanessa Northington Gamble

**Affiliations:** **1**American Medical AssociationChicago, IllinoisUnited States of America; **2**Tuskegee University National Center for Bioethics in Research and Health CareTuskegee, AlabamaUnited States of America

David Wendler and colleagues [ 1] have provided important data to help understand disparities in access to medical research among minorities. It is unfortunate, however, that they draw an unwarranted conclusion from a set of extremely heterogeneous studies. Worse still, by suggesting that the substantial body of research demonstrating how common it is for African Americans to mistrust the health-care system [ 2–4] is wrong, the authors imply that we do not need to come to terms with why this mistrust exists and how it should be addressed by the medical profession.

Wendler et al. note the extreme heterogeneity of the trials included in their study, but they ignore how much this affects the reliability of the meta-analytic techniques they employ. First, the vast majority of the “more than 70,000” patients studied was only involved in survey research—where large differences in response rates between races are not generally seen. Looking only at the clinical trials, the numbers are much smaller and the data become much more difficult to summarize. Among the seven surgical intervention trials studied, two have statistically significant differences between minority enrollment and white enrollment. In one, whites had about 2.7 times greater odds of enrollment than minorities, while in the other, minorities had about 1.6 times greater odds of enrollment than whites. In the ten clinical trials studied, three had statistically different enrollment rates; they, too, had greatly diverging results. For the most part, though, the clinical trials that Wendler et al. examined enrolled so few minority patients (in half of the studies, fewer than 50 minority patients were even asked to enroll), and they are so vastly different in design and objectives that very little information can be reliably gleaned from pooling their results. In fact, one of the largest trials included—the Minority-Based Community Clinical Oncology Program (MBCCOP) cancer trial, which included more than 400 African Americans—was specifically designed to appeal to minority patients, making any assumptions about its generalizability to all medical research extremely suspect. It is well known that meta-analysis is subject to this sort of problem; statistical tricks simply can't account for fundamental differences in studies.

Despite these scientific weaknesses, Wendler et al. are right to conclude that it is inappropriate to focus on changing African Americans' attitudes of mistrust, but not because those attitudes don't exist. Many minorities don't feel welcome and respected within the health-care system. Those who do come in have already crossed a threshold of trust, at least with their individual doctor. Those who don't come in, of course, will never have the opportunity to be asked to enroll in a clinical trial. Instead, the reason it would be inappropriate to focus on changing patient attitudes is because these attitudes of mistrust are based on a history of untrustworthy behavior by the health professions, which must be acknowledged and rectified. In other words, the medical profession should not focus on making minorities be more trusting; we should focus on ensuring that we are becoming trustworthy.
